# 高效液相色谱-蒸发光散射检测法同时测定固态食品中6种稀有糖

**DOI:** 10.3724/SP.J.1123.2023.02014

**Published:** 2023-09-08

**Authors:** Yu LIU, Jiali XING, Jian SHEN, Xiaoli BI, Lingyan MAO, Xiaorong XU, Shufen ZHANG, Yongjiang LOU, Xi WU, Yinghua MU

**Affiliations:** 1.宁波大学食品与药学学院, 浙江 宁波 315211; 1. College of Food and Pharmaceutical Sciences, Ningbo University, Ningbo 315211, China; 2.宁波市产品食品质量检验研究院(宁波市纤维检验所), 浙江 宁波 315048; 2. Ningbo Academy of Product and Food Quality Inspection (Ningbo Fibre Inspection Institute), Ningbo 315048, China

**Keywords:** 高效液相色谱, 蒸发光散射检测器, 稀有糖, 固态食品, high performance liquid chromatography (HPLC), evaporative light-scattering detector (ELSD), rare sugar, solid food

## Abstract

稀有糖在固态食品中的应用量与日俱增,引发了掺假、过量添加等食品安全问题,因此建立固态食品中稀有糖(阿洛酮糖、塔格糖、海藻糖、异麦芽酮糖、赤藓糖醇和甘露糖醇)的检测方法十分重要。本文基于高效液相色谱-蒸发光散射检测器,建立了一种同时检测固态食品中6种稀有糖的分析方法。选用Zorbax Original NH_2_色谱柱(250 mm×4.6 mm, 5 μm)进行分离,流动相为乙腈-水(80∶20, v/v),流速梯度洗脱。蒸发光散射检测系统的漂移管温度设置为50 ℃,雾化器载气为高纯氮,载气流速为1.0 mL/min,压力为275.79 kPa,增益值为3。样品经25 mL水提取,振荡涡旋10 min,分别加入200 μL的乙酸锌溶液和200 μL的亚铁氰化钾溶液净化,4500 r/min离心10 min,取上清液1 mL,过0.22 μm水相滤膜,待上机分析。6种稀有糖在各自范围内线性关系良好,决定系数(*R*^2^)均>0.9985,方法的检出限为0.02~0.60 g/100 g。以空白固态食品样品为基质,在3种加标水平下,6种稀有糖的回收率为92.6%~103.2%,相对标准偏差为0.7%~4.4%。将该方法用于分析实际固态食品样品中6种稀有糖的含量,结果显示,实际成分和食品标签所示基本一致。本研究建立的方法前处理操作简单,重复性好,方法快速、灵敏、准确,可应用于日常监测固态食品中6种稀有糖的含量,同时为稀有糖的检测提供了一种具有广阔前景的技术手段。

## Abstract (en)

Excessive sugar consumption is associated with metabolic health problems. Rare sugars are gradually being used as substitutes for sugar, and their consumption is increasing daily, raising food-safety issues such as false advertising, adulteration, and overdosing. The determination of rare-sugar compounds has attracted considerable attention in recent years. However, no standard method for the simultaneous determination of six rare sugars (allulose, tagatose, trehalose, isomaltulose, erythritol, and mannitol) in solid foods is available. Therefore, establishing a suitable analytical method for these sugars is necessary. In this study, high performance liquid chromatography coupled with evaporative light-scattering detection was used to determine rare sugars in solid foods. The optimum chromatographic and detector conditions were determined by evaluating the instrument parameters. Analysis was carried out on a Zorbax Original NH_2_ column (250 mm×4.6 mm, 5 μm) via flow-rate gradient elution (0-15 min, 1.0 mL/min; 15-18 min, 1.0-2.0 mL/min; 18-25 min, 2.0 mL/min) with acetonitrile-water (80∶20, v/v) as the mobile phase. Sharp and symmetric chromatographic peaks were obtained under these conditions. The resolutions for all the six rare sugars were greater than 1.5. Optimization of the evaporative light-scattering detector was extremely important to the responses of the rare-sugar compounds. The two most significant parameters were the nebulizer carrier gas flow rate and drift tube temperature. The detection system was operated under the following conditions: the drift tube temperature was set to 50 ℃, the nebulizer carrier gas was high-purity nitrogen, the carrier gas flow rate was 1.0 mL/min, the nitrogen pressure was regulated to 275.79 kPa, and the gain factor was set to 3. The sample was extracted with 25 mL of water, shaken and vortexed for 10 min, purified with 200 μL of zinc acetate solution and 200 μL of potassium ferricyanide solution, and centrifuged at 4500 r/min for 10 min. Next, 1 mL of the supernatant was passed through a 0.22 μm aqueous-phase filter membrane, and the filtrate obtained was analyzed using the evaporative light-scattering detector. The six rare sugars were quantitatively analyzed using the external standard method and showed good linearity with coefficients of determination (*R*^2^) greater than 0.9985. The limits of detection and quantification were 0.020-0.60 and 0.60-1.8 g/100 g, respectively. In addition, when blank solid food samples were spiked with the analytes at three levels, the average recoveries of the six rare sugars were 92.6%-103.2%, with relative standard deviations (RSDs) of 0.7%-4.4%. An RSD of <5% indicated that the method had good precision. Interference experiments were performed to determine whether the sugars and artificial sweeteners commonly found in solid foods affected the targets. The method established in this study was used to analyze the contents of the six rare sugars in actual solid food samples. The experimental results showed various levels of rare glycoconjugates in different solid foods. Moreover, the actual compositions and labeled of rare glycoconjugates in the solid foods were generally consistent. The proposed method features simple operation, rapid results, high sensitivity, and good reproducibility; thus, it meets the requirements for the detection of the six rare sugars in solid foods. It also provides technical support for the development of methodological standards and detection limits for rare sugars in Chinese foods. The results of this study are of great relevance for the daily monitoring of the levels of the six rare sugars in solid foods.

糖(sugar)主要由C、H、O 3种元素组成,是多羟基醛或多羟基酮及其缩聚物和某些衍生物的总称,被广泛应用于食品甜味剂来提升甜度和风味。目前,市场上常见的甜味剂为蔗糖、葡萄糖和人工甜味剂等^[1]^。然而过量摄入甜味剂会引发多种健康问题,例如2型糖尿病、肥胖症和心血管疾病^[2,3]^。而且人工甜味剂的安全性目前存在一定争议^[4]^。因此,为了减少糖和人工甜味剂的消耗,稀有糖得到越来越多的关注。

稀有糖被定义为自然界中少量存在的单糖及其衍生物^[5]^。大量研究报道了稀有糖的有益功能^[6⇓-8]^,例如不引起龋齿、减轻体重、控制血糖等。近年来,赤藓糖醇和甘露糖醇在全球范围内代糖销售市场火热^[9]^,阿洛酮糖和塔格糖作为新型糖类成为最有前途的蔗糖替代品^[10]^,异麦芽酮糖和海藻糖是广泛研究的稀有二糖^[11]^,国外相关机构已将这6种稀有糖视为功能性甜味剂,允许加入食品中。然而,以下原因造成了稀有糖的食品安全问题:(1)食品添加剂联合专家委员会(Joint FAO/WHO Expert Committee on Food Additives)、美国食品药品监督管理局(Food and Drug Administration)、欧盟(European Union)和澳新食品标准局(Food Standards Australia New Zealand)都未指定这6种稀有糖每日可接受摄入量(acceptable daily intake, ADI)^[12,13]^。(2)需要大量添加或复合添加稀有糖才能达到一定的甜度^[14⇓-16]^,这可能会引发过量食用稀有糖导致胃肠道反应(如腹泻、饱胀)并扰乱血糖水平^[17]^。部分食品厂商用廉价或无营养价值的非糖类物质(玉米淀粉、麦芽糊精等)或含有杂质的低质量工业糖来掺假添加,夸大宣传其功效来误导消费者购买^[18]^。(3)这6种稀有糖同时检测的方法尚未成熟。因此,为了安全合理地使用稀有糖,亟须开发一种高效、灵敏,且可以同时检测分析食品中6种稀有糖的检测方法。

常见的糖类分析检测方法有气相色谱法(GC)^[19]^、气相色谱-质谱法(GC-MS)^[20]^、毛细管电泳法(CE)^[21]^、高效阴离子交换色谱-脉冲安培检测法(HPAEC-PAD)^[22]^和高效液相色谱(HPLC)联用不同检测器的方法,如质谱(MS)^[23]^、紫外检测器(UV)^[24]^、示差折光检测器(RI)^[25]^、蒸发光散射检测器(ELSD)^[26]^。GC和GC-MS需要衍生化,步骤复杂、耗时。CE可以快速分析塔格糖^[27]^,然而没有关于其他稀有糖的检测报道。HPAEC-PAD使用氢氧化物水溶液作为流动相会引起稳定性问题。此外,基于HPLC分离系统,MS仪器昂贵,前处理涉及复杂的衍生化过程。由于糖类无发色团,UV分析受到限制。相比之下,RI和ELSD已被广泛应用于分析食品中的糖,无需衍生化即可进行快速分析^[26,28]^。然而,RI检测器不兼容梯度洗脱,灵敏度易受温度影响。ELSD不存在上述这些情况,要求流动相必须有良好的挥发性。目前,尚无采用HPLC-ELSD对6种稀有糖进行同时检测的相关报道。

因此,在这项研究中,开发了一种新颖的HPLC-ELSD方法,经过方法优化可以在25 min内快速同时准确定性定量分离固态食品中6种稀有糖,也为我国食品中制定稀有糖检测方法标准和限量标准提供技术支持。

## 1 实验部分

### 1.1 仪器、试剂与材料

Agilent 1260 Infinity Ⅱ高效液相色谱仪和蒸发光散射检测器(美国Agilent公司); ME-204电子分析天平(精度为0.000 1 g,上海梅特勒-托利多仪器有限公司); X1R高速离心机(美国Thermo公司); Vortex 3自动漩涡混合器、IKA-Vortex 2多功能涡旋混合器(德国IKA公司);一次性无菌注射器(上海金塔医用器材有限公司); Milli-Q Academic超纯水仪(电阻率为18.2 MΩ·cm,美国Millipore公司); KS-300EI超声波清洗仪(宁波科生超声设备有限公司); GFAM00140移液枪(法国GILSON公司); 0.22 μm滤膜(美国Waters公司)。

阿洛酮糖(allulose,纯度>98%)、塔格糖(tagatose,纯度>98%)、海藻糖(trehalose,纯度>98%)、异麦芽酮糖(isomaltulose,纯度>98%)、赤藓糖醇(erythritol,纯度>98%)、甘露糖醇(mannitol,纯度>98%), HPLC级,均购自上海麦克林生化科技股份有限公司。

乙腈(HPLC级,纯度>99.9%)购自上海安谱实验科技股份有限公司;乙酸锌(C_4_H_6_O_4_Zn·2H_2_O)、亚铁氰化钾(K_4_Fe(CN)_6_·3H_2_O)(均为分析纯)购自上海默克化工技术有限公司。

实验用固态食品:面包、口香糖、巧克力、饼干、糖果均购自网络,藕粉购自宁波大型超市。

### 1.2 溶液配制

标准储备液:分别准确称取各种稀有糖标准品各0.1 g(精确至0.0001 g)于10 mL容量瓶中,用超纯水定容至刻度,配合超声加速溶解,配制成各质量浓度为10 mg/mL的标准储备液;分别吸取阿洛酮糖、塔格糖、赤藓糖醇200 μL,甘露糖醇300 μL,异麦芽酮糖、海藻糖600 μL于10 mL容量瓶中,用超纯水定容至刻度,转移至10 mL棕色进样瓶中,得到含有不同浓度的混合标准储备液,密封后置于4 ℃冰箱中保存备用。

标准工作液:用超纯水将混合标准储备液逐级稀释配制成质量浓度分别为0.03、0.06、0.08、0.10、0.12、0.15、0.20 mg/mL的6种稀有糖的混合标准工作液。

乙酸锌溶液(0.219 g/mL):称取乙酸锌21.9 g,加冰醋酸3 mL,加水溶解并稀释至100 mL。

亚铁氰化钾溶液(0.106 g/mL):称取亚铁氰化钾10.6 g,加水溶解并稀释至100 mL。

### 1.3 样品前处理

称取0.5 g样品至50 mL螺口尖底离心管中,加入25 mL水(样品处理用水为一级水),振荡涡旋10 min,分别加入200 μL的乙酸锌溶液和200 μL的亚铁氰化钾溶液,用水定容至刻度,充分摇匀,4500 r/min离心10 min,移取上清液1 mL,过0.22 μm水相滤膜,待HPLC-ELSD分析。

### 1.4 分析条件

色谱条件:Zorbax Original NH_2_色谱柱(250 mm×4.6 mm, 5 μm,美国Agilent公司);柱温为35 ℃;流动相:乙腈-水(80∶20, v/v);进样体积20 μL;流速梯度洗脱程序:0~15 min, 1.0 mL/min; 15~18 min, 1.0~2.0 mL/min; 18~25 min, 2.0 mL/min; 15~25 min, 2.0 mL/min。

ELSD条件:漂移管温度为50 ℃,雾化器流速为1.0 mL/min(载气为高纯氮),载气压力为275.79 kPa,增益值为3。

### 1.5 数据处理

采用SPSS 25统计软件进行相关性分析和数据处理,采用Origin 2022b和Microsoft Excel进行图表绘制。

## 2 结果与讨论

### 2.1 前处理条件的优化

#### 2.1.1 提取剂类别的优化

不同提取剂会影响稀有糖的提取效果。为了确定提取剂,本实验比较了水、乙腈和不同体积分数(80%、60%、40%和20%)的乙腈水溶液对6种稀有糖提取效果的影响。结果表明,对比乙腈水溶液和乙腈,水对稀有糖的提取效果最显著(图1),6种稀有糖的回收率(94.6%~101.4%)明显提升,这与Georgelis等^[29]^的研究结果一致,故选择水作提取剂。

**图 1:**
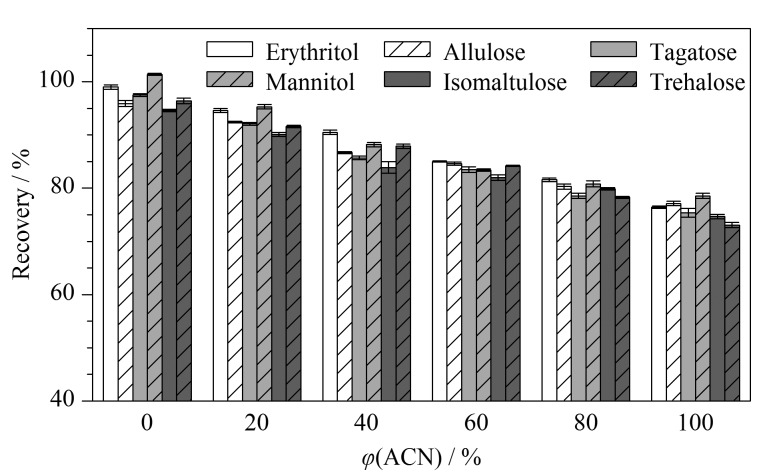
不同提取剂对6种稀有糖回收率的影响(*n*=6)

#### 2.1.2 净化剂用量的优化

固态食品中有蛋白质、明胶、脂肪等物质,未经净化剂处理直接进样会影响稀有糖的检测分析。因此本实验参考GB 5009.8-2016《食品安全国家标准食品中果糖、葡萄糖、蔗糖、麦芽糖、乳糖的测定》^[30]^将乙酸锌和亚铁氰化钾作为净化剂。同时,考察了乙酸锌和亚铁氰化钾各自的用量(100~300 μL,添加时两者的用量为等量)对6种稀有糖回收率的影响。由图2可以看出,乙酸锌和亚铁氰化钾各为200 μL时,稀有糖的回收率相对较高(97.41%~101.4%),故乙酸锌和亚铁氰化钾用量确定为200 μL。

**图 2:**
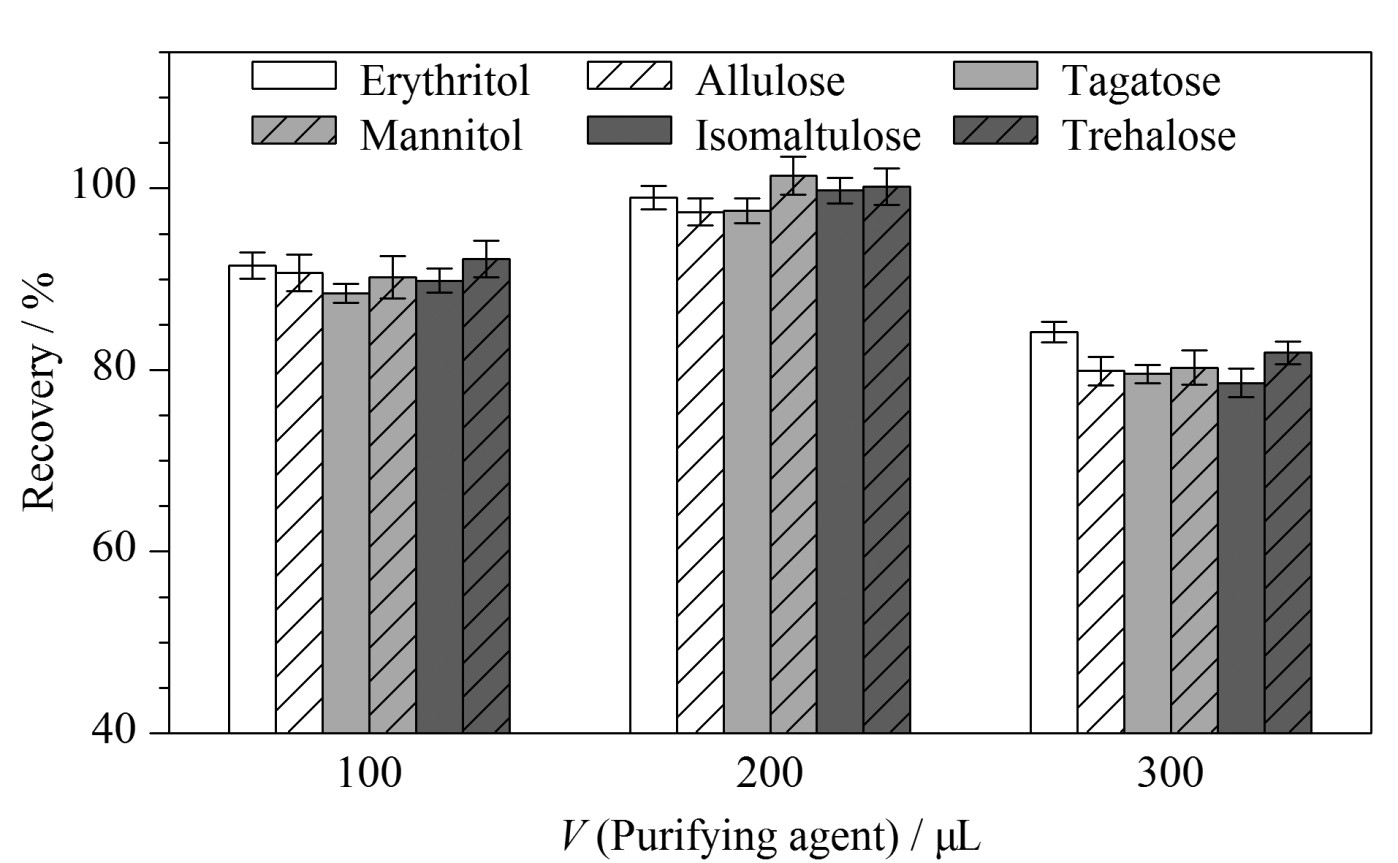
不同用量的净化剂对6种稀有糖回收率的影响(*n*=6)

### 2.2 仪器条件的优化

#### 2.2.1 色谱条件的优化

检测糖类常用的检测器为蒸发光散射检测器^[31]^和示差折光检测器^[32]^,但示差折光检测器极易受温度影响,且不适用于梯度洗脱。蒸发光散射检测器受温度影响小,基线稳定,灵敏度高,可以结合梯度洗脱,因此在稀有糖检测分析中选择了蒸发光散射检测器。

分别选择不同比例(85∶15、80∶20和75∶25)的乙腈-水作为流动相,比较3个流动相比例对分离效果的影响。结果发现,水的比例增加会缩短保留时间,但是基线噪声比较大;乙腈的比例增加会增加保留时间。因此,本实验选择乙腈-水(80∶20, v/v)作为流动相。

取质量浓度均为0.1 mg/mL的6种稀有糖混合标准溶液,在保持浓度、柱温、进样量等参数一致的条件下,比较Agilent Zorbax Original NH_2_(250 mm×4.6 mm, 5 μm)和ChromCore HILIC-Amide(250 mm×4.6 mm, 5 μm)这2种色谱柱对6种稀有糖的分离效果。结果显示,NH_2_柱分离效果优于Amide柱,这是由于NH_2_柱与不同糖分子所形成氢键的结合力强弱不同,在分离时更具有优势^[33]^,因此选择NH_2_柱作为分析柱。同时优化柱温和流速梯度洗脱程序,确定柱温为35 ℃;确定的洗脱程序见1.4节。

#### 2.2.2 ELSD的优化

ELSD最关键的两个参数是漂移管温度和雾化器载气流速^[34]^。雾化器载气流速决定了雾化过程中形成的液滴大小,从而影响检测器的信噪比(signal to noise ratio, *S/N*)^[33]^。漂移管温度影响检测器的响应,随着温度的升高,流动相蒸发趋于完全,响应会更好,但过高或过低的温度会降低*S/N*^[35]^。考虑到洗脱液挥发性、流速、雾化器载气流速以及分析物挥发性等对稀有糖检测结果的影响,合理设置漂移管温度可以有效提高分析结果的灵敏度。雾化器载气流速和漂移管温度几乎不影响稀有糖的保留时间。因此,在其他参数一致的条件下,先固定载气流速为1.2 mL/min,对6组漂移管温度(30、40、50、60、70和80 ℃)进行考察;筛选出最佳漂移管温度后对4组载气流速(1.0、1.2、1.4和1.6 mL/min)进行考察,结果如图3显示。

**图 3:**
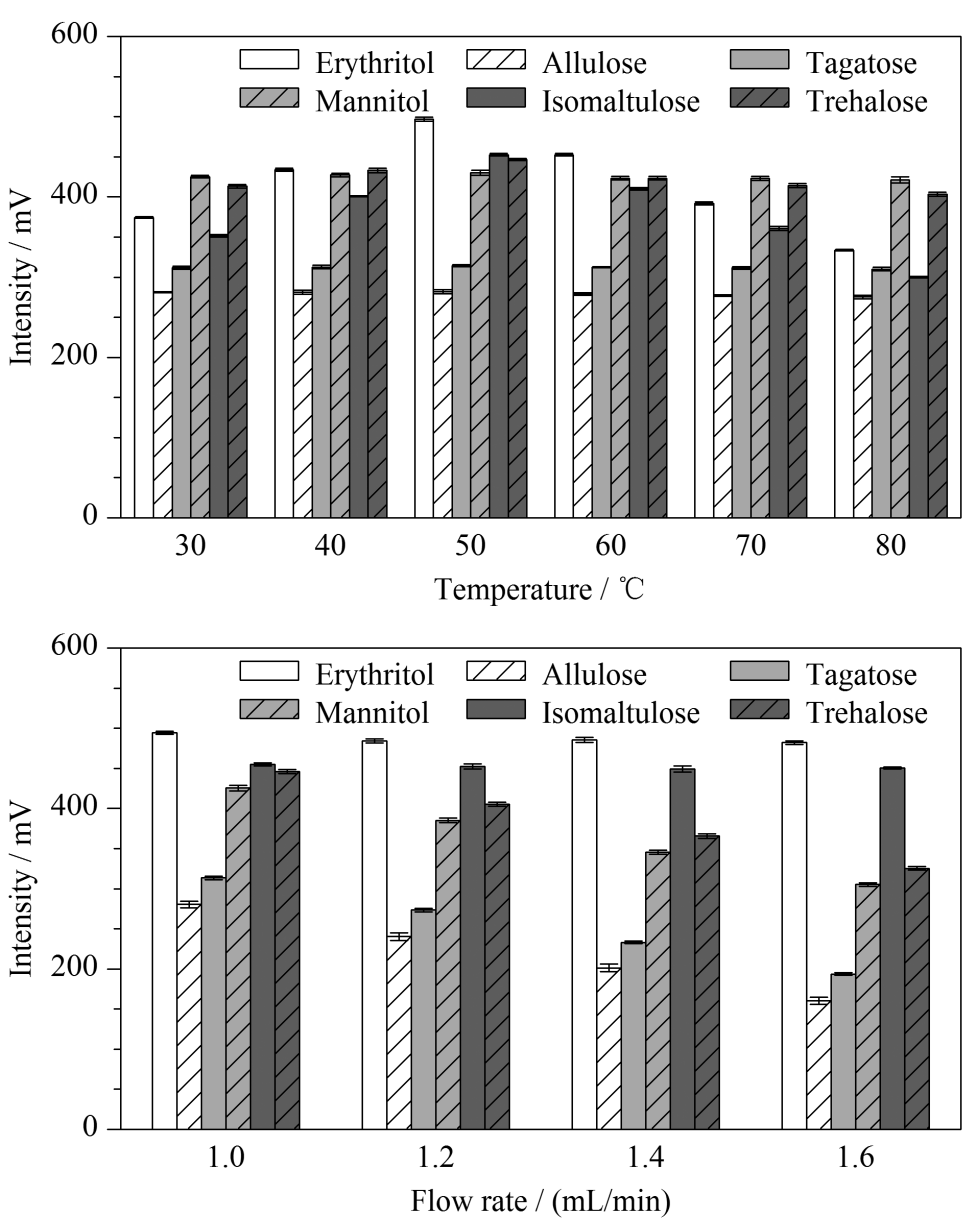
不同漂移管温度和载气流速对6种稀有 糖响应的影响(*n*=3)

优化漂移管温度时,赤藓糖醇、异麦芽酮糖和海藻糖的响应变化明显(*P*<0.05),对比其他5组,50 ℃时,赤藓糖醇、异麦芽酮糖和海藻糖(496.82、452.70和446.66 mV)的响应明显上升;其余3种稀有糖的响应变化不明显的可能原因是它们的挥发性小,对温度不敏感。优化载气流速时,阿洛酮糖、塔格糖、甘露糖醇和海藻糖的响应变化明显(*P*<0.05),对比其他3组,在1.0 mL/min时4种稀有糖(280.35、313.51、425.44和446.01 mV)的响应明显最好;赤藓糖醇和异麦芽酮糖的响应不随载气流速变化的可能原因是1.0 mL/min的载气流速已经足够使这两种稀有糖产生的液滴大小达到最大,增加载气流速不会对液滴大小产生显著影响。赤藓糖醇和甘露糖醇响应的变化也与丁洪流等^[33]^研究一致:适宜的漂移管温度和载气流速有利于提高赤藓糖醇和甘露糖醇的响应。因此,最终确定漂移管温度为50 ℃,雾化器载气流速为1.0 mL/min。在最优条件下得到的6种稀有糖色谱图见图4,显示6种稀有糖在25 min内能够有效分离,分离度均大于1.5,可达到分析要求。

**图 4:**
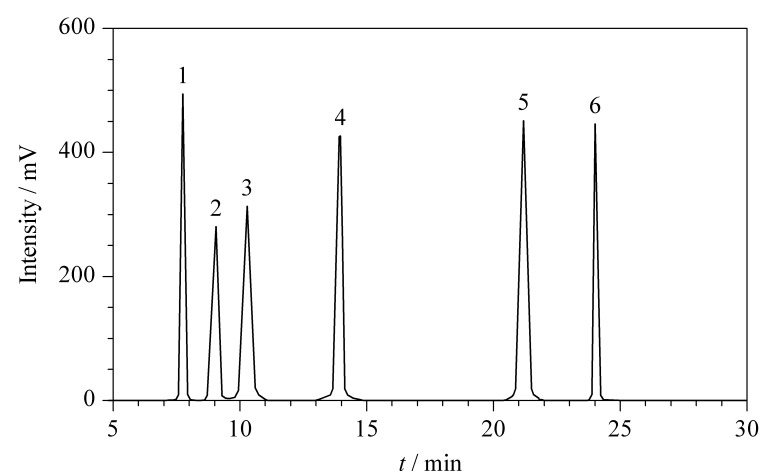
6种稀有糖混合标准溶液的色谱图

### 2.3 方法学验证

#### 2.3.1 线性范围、检出限及定量限

以超纯水为溶剂,配制质量浓度范围为0.06~0.6 mg/mL的6种稀有糖混合标准溶液直接进样检测。以稀有糖峰面积(*Y*)为纵坐标,质量浓度为横坐标(*X*, mg/mL),绘制校准曲线(表1)。结果显示,6种稀有糖的线性关系良好,决定系数(*R*^2^)均>0.9985,以*S/N*=3和*S/N*=10分别确定方法的检出限(LOD)和定量限(LOQ),分别为0.20~0.60 g/100 g和0.60~1.8 g/100 g。

**表 1:** 6种稀有糖的线性范围、回归方程、决定系数、检出限及定量限

Compound	Linear range/(mg/mL)	Regression equation	R^2^	LOD/(g/100 g)	LOQ/(g/100 g)
Erythritol	0.06-0.2	y=1.330x+12.13	0.99977	0.20	0.60
Allulose	0.06-0.2	y=1.288x+11.87	0.99975	0.30	0.60
Tagatose	0.06-0.2	y=1.257x+12.11	0.99901	0.20	0.60
Mannitol	0.09-0.3	y=1.289x+12.53	0.99907	0.45	0.90
Isomaltulose	0.18-0.6	y=1.156x+12.20	0.99854	0.60	1.8
Trehalose	0.18-0.6	y=1.096x+12.07	0.99959	0.50	1.8

*y*: peak area; *x*: mass concentration, mg/mL.

#### 2.3.2 加标回收率与精密度

在进行加标回收试验前,对空白固态食品进行分析,确保不含任何干扰成分。同时,通过比较混合标准溶液、空白基质溶液和空白基质加标溶液的色谱图(见图5),评估方法是否受到样品基质的干扰。

**图 5:**
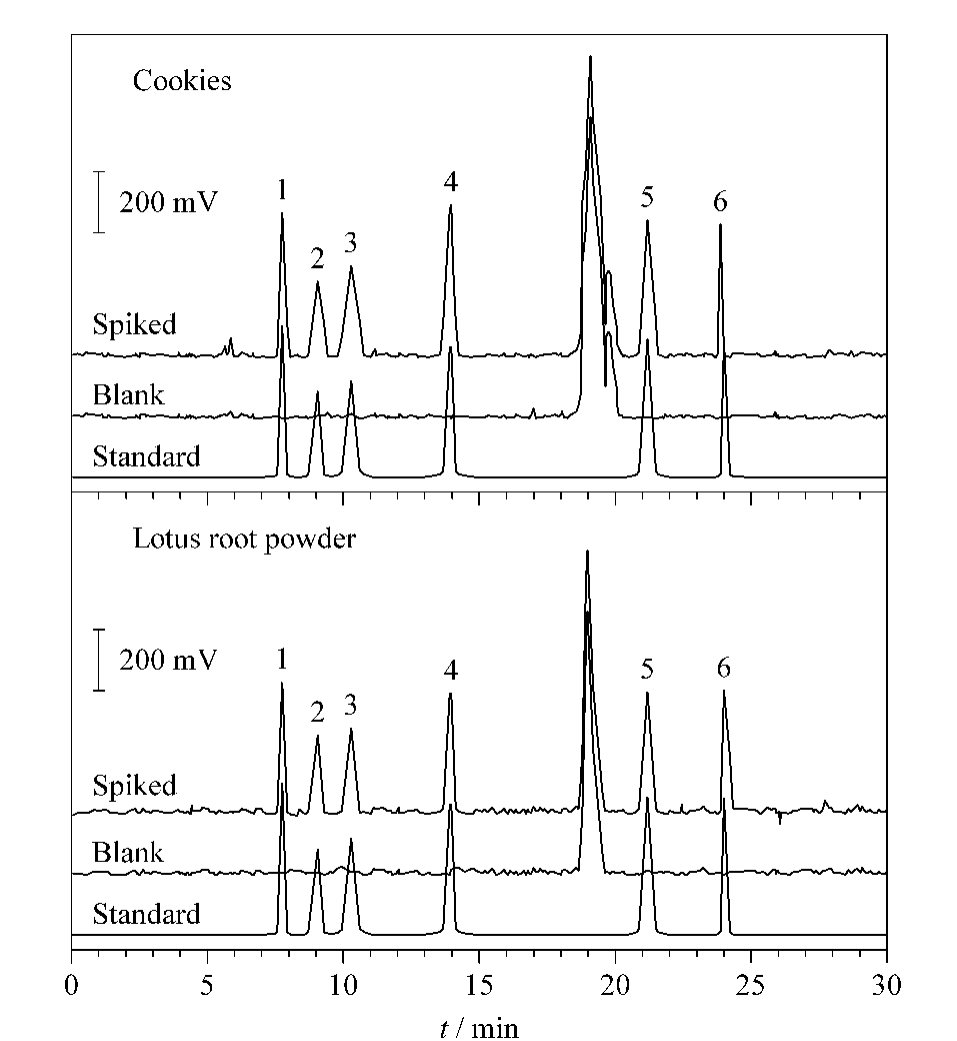
混合标准溶液、空白基质溶液和空白基质 加标溶液的色谱图

以空白饼干样品为基质,按1.3节处理,分别添加低、中、高3个不同水平的混合标准溶液进行测定。每个水平重复测定6次,计算平均回收率和相对标准偏差(RSD)来评估方法的准确性和精密度。结果如表2所示,6种稀有糖的平均加标回收率为92.6%~103.2%, RSD为0.7%~4.4%,表明该方法性能良好,能够满足实际样品检测要求。

**表 2:** 6种稀有糖的加标回收率和精密度(*n*=6)

Compound	Low			Medium			High	
	Recovery/%	RSD/%		Recovery/%	RSD/%		Recovery/%	RSD/%
Erythritol	102.0	1.4		96.9	3.2		95.3	1.3
Allulose	94.6	2.9		94.2	3.4		93.5	2.4
Tagatose	95.0	0.7		92.6	2.8		93.3	2.3
Mannitol	103.2	1.1		100.8	2.0		93.9	4.4
Isomaltulose	98.4	3.9		98.4	2.8		96.5	2.6
Trehalose	100.4	1.7		99.7	2.3		101.0	3.8

Low, medium and high spiked levels: for erythritol, allulose and tagatose, 0.6, 1, and 2 g/100 g; for mannitol, 0.9, 1.5, and 6 g/100 g; for isomaltulose and trehalose, 1.8, 3, and 6 g/100 g.

#### 2.3.3 干扰试验

为了防止固态食品自身可能含有的糖和人工甜味剂干扰实验结果,从而出现假阳性现象。因此,实验对配制的包含6种稀有糖、5种常见糖(葡萄糖、果糖、蔗糖、乳糖、麦芽糖)和3种人工甜味剂(三氯蔗糖、阿斯巴甜、甜蜜素)的混合溶液进行分析。如图6所示,其中4种常见糖可与本文分析的6种稀有糖基线分离,其余1种常见糖及3种人工甜味剂未出峰,说明常见的5种糖和人工甜味剂对本方法均无任何干扰。

**图 6:**
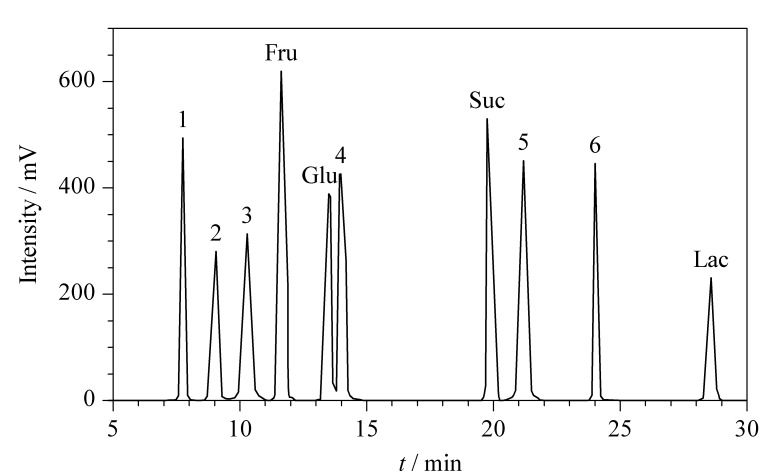
干扰试验色谱图

### 2.4 与其他方法的比较

本研究与近年使用HPLC-ELSD测定同种稀有糖的方法进行了简单对比(表3)。结果显示,本研究对比其他文献涉及的稀有糖种类新颖,数量较多,在前处理方面对比刘文全^[37]^检测饮料中的阿洛酮糖和Koh等^[31]^检测固态食品中的赤藓糖醇和甘露糖醇,操作简单、省时。同时与其他文献相比,阿洛酮糖、赤藓糖醇和甘露糖醇具有较低的检出限。

**表 3:** 与其他文献方法的比较

Matrices	Rare sugar counts	Compound	Pre-treatment time/min	LOQ/ (mg/mL)	Ref.
Bread, chocolate, chewing gum, cookie, candy, lotus root powder	6	erythritol, allulose, tagatose, mannitol, isomaltulose, trehalose	20	0.06-0.18	this work
Honey	1	allulose	-	1.0	[36]
Candy, chewing gum, jelly, chocolate, snack, processed chocolate products	2	erythritol, mannitol	65	1.3-1.7	[31]
Beverage	1	allulose	30	0.060	[37]
Food product	2	erythritol, mannitol	25	0.15-0.24	[33]

### 2.5 实际样品的检测

应用本实验建立的方法对购买的面包、巧克力、口香糖、饼干、糖果、藕粉6类标签中标注含稀有糖的固态食品样品进行检测,每份样品平行测定3次。典型固态食品的色谱图见图7。

**图 7:**
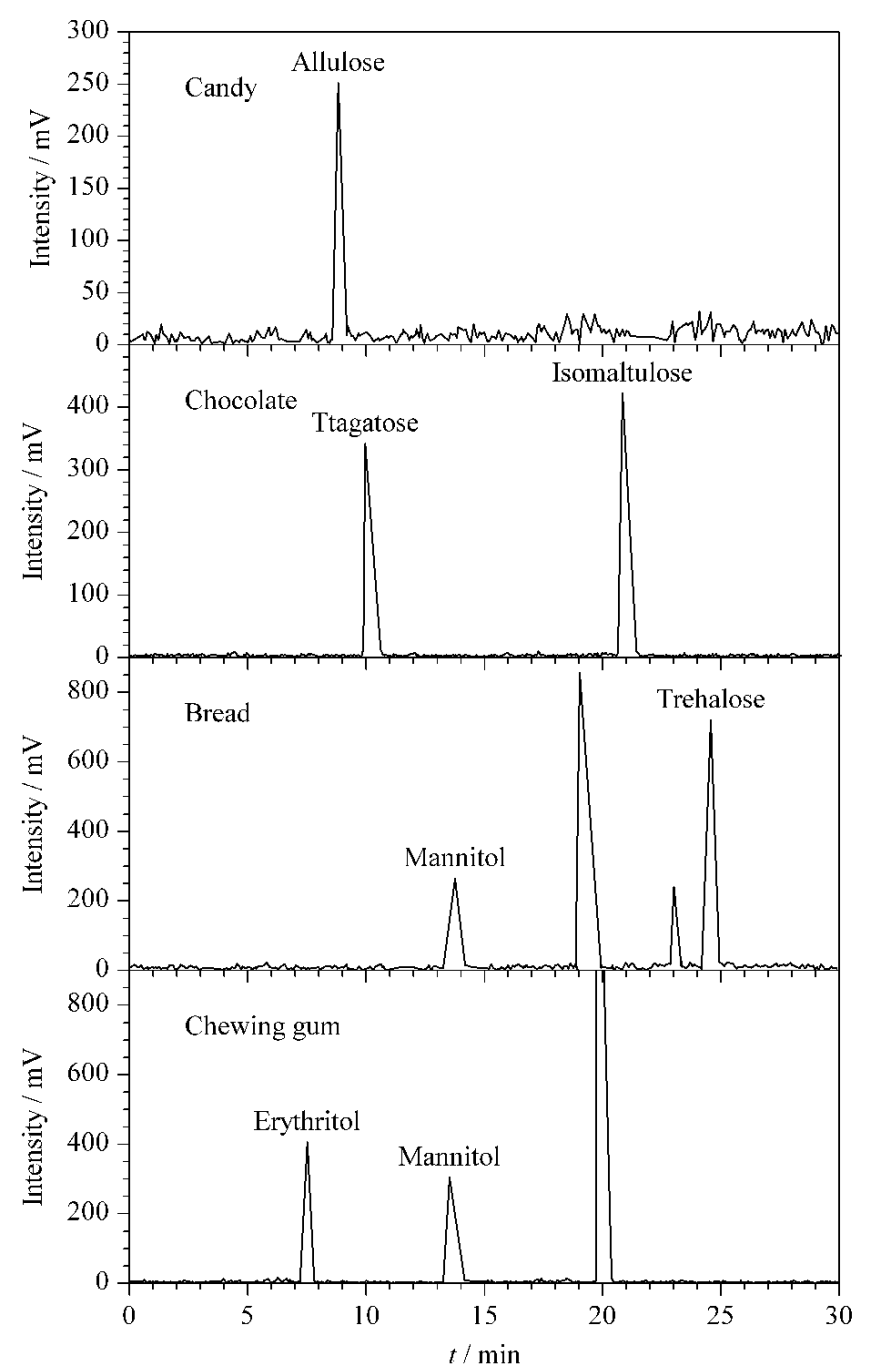
典型固态食品的色谱图

结果显示,巧克力、饼干和糖果含有10.9~29.5 g/100 g(RSD为0.1%~1.2%)的稀有糖,含糖量为10.9%~29.5%;面包、口香糖和藕粉含有1.2~9.4 g/100 g(RSD为0.3%~1.7%)的稀有糖,含糖量为1.2%~9.4%。其中甘露糖醇在口香糖的含量与Koh等^[31]^研究的含量在同一量级。检出的稀有糖符合样品标签中标注的稀有糖。证明了该方法可以用来同时分析固态食品中的稀有糖。

## 3 结论

本研究成功建立了一种能够在25 min内完全分离6种稀有糖的HPLC-ELSD方法。通过对实际样品的检测分析,发现固态食品标签中声明的稀有糖与实际成分基本一致。然而,部分固态食品中的稀有糖含量超过10%,食用后可能导致胃肠道反应。因此,我们建议在商品标签中明确标示稀有糖的含量及可能出现的健康问题,从而向消费者提供准确的信息去选择合适的商品。该方法为固态食品中稀有糖的准确分析提供了可靠的手段,也为糖类食品的质量控制和安全评价提供了科学依据。
